# Decision-making in postoperative chemotherapy for ovarian metastasis from colorectal cancer: a retrospective single-center study

**DOI:** 10.1186/s12957-022-02498-1

**Published:** 2022-02-01

**Authors:** Shunsuke Hamasaki, Yosuke Fukunaga, Satoshi Nagayama, Yoshiya Fujimoto, Takashi Akiyoshi, Toshiya Nagasaki, Masashi Ueno

**Affiliations:** Division of Colorectal Surgery, Department of Gastroenterological Surgery, Cancer Institute Hospital, 3-10-6 Ariake, Koto-ku, Tokyo, 135-8550 Japan

**Keywords:** Colorectal cancer, Ovarian metastasis, Chemotherapy, Long-term outcome, Chemotherapeutic option, Krukenberg tumor, Oophorectomy

## Abstract

**Background:**

Ovarian metastases from colorectal cancer are relatively uncommon, and no consensus has been reached regarding resection of metastases or chemotherapy before and after surgery. We evaluated the clinicopathological characteristics of ovarian metastases from colorectal cancer and the impact of metastatic resection. We also performed a comparative analysis to clarify the prognostic impact of metastatic resection and the choice of chemotherapy before and after surgery.

**Methods:**

Between 2006 and 2014, 38 patients at our institution underwent resection of ovarian metastases from colorectal cancer. Clinicopathological data were extracted from the patients’ records and evaluated with respect to the long-term outcome. For 15 patients with metachronous ovarian metastases who received chemotherapy until immediately before resection, we compared the prognosis with and without changes in the regimen after resection.

**Results:**

The 5-year overall survival rate was 19.9%, and the median survival duration was 27.2 months. The survival rate in the R0 resection group (*n* = 8) was significantly better than that in the R1/2 resection group (*n* = 30) (*P* = 0.0004). Patients without peritoneal dissemination (*n* = 15) or extra-ovarian metastases (*n* = 31) had a significantly better prognosis than those with peritoneal dissemination (*n* = 23) or extra-ovarian metastases (*n* = 7) (*P* = 0.040 and *P* = 0.0005, respectively). The progression-free survival and median survival times of patients who resumed chemotherapy after resection without a change in their preoperative regimen were 10.2 months and 26.2 months, respectively, while those among patients with a change in their regimen before resection versus after resection were 11.0 months and 18.1 months, respectively. The difference between the two groups was not statistically significant (progression-free survival time and median survival time: *P* = 0.52 and *P* = 0.48, respectively).

**Conclusions:**

Patients who underwent R0 resection of ovarian metastases clearly had a better prognosis than those who underwent R1/2 resection. Additionally, a poor prognosis was associated with the presence of peritoneal dissemination and extra-ovarian metastases. The data also suggested that resumption of chemotherapy without changing the regimen after resection could preserve the next line of chemotherapy for future treatment and improve the prognosis.

## Background

Colorectal cancer (CRC) is the second most frequent cancer and the most common cause of cancer death in Japanese women. While the prognosis of early-stage CRC is relatively good, with a 5-year survival rate of 91.6% for stage I disease, that of metastatic CRC is remarkably poor; in patients with stage IV disease, it is only 18.8%.

Ovarian metastases from CRC are relatively uncommon, occurring in 2.1% to 13.6% of female patients after colorectal resection [1], but the prognosis is poor. Although liver or lung metastasis from CRC can be resected with good long-term outcomes, it is unclear whether the resection of ovarian metastasis improves the prognosis.

With the remarkable advances in chemotherapy for CRC the improvements in outcomes for patients with distant metastases have been substantial. However, compared with metastases in other organs, ovarian metastasis is less responsive to chemotherapy [2, 3], such that surgical intervention in conjunction with chemotherapy remains an attractive option to improve the prognosis of these patients. However, few reports have investigated the prognostic effects of surgical resection of ovarian metastases and the performance of chemotherapy before and after surgery, and no consensus has been reached. In particular, no reports have investigated the effect of the choice of resuming chemotherapy on the prognosis after resection.

In this study, we evaluated the clinicopathological characteristics of ovarian metastasis from CRC and the prognostic impact of the resection of ovarian metastasis. We also performed a comparative analysis to clarify the prognostic impact of resection of ovarian metastasis and the choice of chemotherapy regimen to resume after resection.

## Methods

### Patients

This was a retrospective study of 38 patients who underwent resection of ovarian metastases from CRC between June 2006 and December 2014. All patients had pathologically confirmed metastasis from CRC. The data were extracted from the patients’ medical records and from the database of the Cancer Institute Hospital.

This study was performed in accordance with the Declaration of Helsinki, and it complied with the study protocol and the Ethical Guidelines for Medical and Health Research Involving Human Subjects. This study was approved by the institutional review board of the Cancer Institute Hospital (approval code 0908). The protocol summary was described on the hospital website, and the patients were provided with the opportunity to opt-out. Therefore, no new consent for this study was required from the patients.

Clinical variables included age, presenting symptoms, menopausal status, primary tumor site and the presence of other metastatic sites, chronological status of the metastases (simultaneous or metachronous), tumor size, side of metastasis, operation time, and complete resection. The pathological findings of the primary tumor, including histology, depth of invasion, and lymph node metastasis, were also investigated. For each item, the 5-year survival rate and the median survival time (MST) were derived using the Kaplan–Meier method and verified by univariate analysis.

In addition, the patients whose chemotherapy was the same as before ovarian metastasis resection were compared with patients whose chemotherapy was changed after resection. For 15 patients with metachronous ovarian metastases who received chemotherapy until immediately before resection, we compared the prognosis with and without changes in the regimen after resection. For these comparisons, progression-free survival (PFS) was used in addition to MST.

### Patient follow-up

After primary resection, standard chemotherapy was administered according to the patient’s preference, current guidelines, and the decision made by a multidisciplinary team. Postoperative follow-up was conducted every 6 months and included a physical examination, determination of serum levels of tumor markers, and chest and abdominal computed tomography (CT) scans. A colonoscopy was performed every 1 to 2 years. Response criteria were defined according to the Response Evaluation Criteria in Solid Tumors (RECIST) guidelines, version 1.1.

### Statistical analysis

Clinical and pathological variables were compared by cross-table analysis using Fisher’s exact test. Survival was analyzed by the Kaplan–Meier method and the results were compared using the log-rank test. Potential variables were verified by univariate analysis using logistic regression. A *P* value of < 0.05 was considered to indicate statistical significance. All calculations were performed using JMP software version 12 (SAS Institute Inc., Cary, NC, USA).

## Results

### Patient characteristics

The median age at diagnosis was 50 years (range, 25–81 years); 16 patients (42.1%) were < 49 years of age at diagnosis. Twenty-eight patients (73.7%) were pre-menopausal and 10 (26.3%) were post-menopausal. The most common symptom at presentation was abdominal distension in 15 patients (39.5%), followed by abdominal pain in 4 patients (10.5%). Ovarian metastases were diagnosed by CT scan during the follow-up period in 19 patients (50.0%) without any symptoms. The median serum tumor marker levels at ovarian resection were 31.2 ng/ml for carcinoembryonic antigen (range, 1.6–2726.0 ng/ml), 20.2 ng/ml for CA19-9 (range, < 2.0–3782.9 ng/ml), and 97.2 ng/ml for CA125 (range, 6.6–1616.3 ng/ml).

### Clinical and pathological characteristics of the primary tumor

Primary tumors were located in the proximal colon (cecum to transverse colon, including the appendix) in 12 patients (31.6%), the distal colon (descending and sigmoid colon) in 19 patients (50.0%), and the rectum in 7 patients (18.4%). The depth of invasion of the primary cancer was T2 in 1 patient (2.6%), T3 in 11 patients (28.9%), T4 in 24 patients (63.2%), and unknown in 2 patients (5.3%). Lymph node metastases were detected in 29 patients (76.3%). The histological type of the adenocarcinoma was well-differentiated in 7 patients (18.4%), moderately differentiated in 24 patients (63.1%), mucinous in 2 patients (5.3%), poorly differentiated in 2 patients (5.3%), and unknown in 3 patients (7.9%).

### Clinical characteristics of ovarian metastasis

Synchronous ovarian metastases occurred in 9 patients (23.7%) and metachronous ovarian metastases occurred in 29 (76.3%). In the latter group, the median interval from the primary surgery to the diagnosis of ovarian metastasis was 13.6 months (range, 3.3–66.3 months) and that to the resection of ovarian metastasis was 18.7 months (range, 4.0–69.2 months).

### Surgical resection of ovarian metastases

Bilateral ovarian resection was performed in 30 patients. Within this group, bilateral ovarian metastases occurred in 14 patients (46.7%), including 5 who were diagnosed with unilateral metastasis preoperatively. The median size of ovarian tumors was 12.0 cm (range, 2–41 cm). R0 resection was achieved in eight patients (21.1%). Of these 8 patients, 7 (18.4%) presented with metastasis confined to the ovaries; the remaining 31 patients had simultaneous metastases to other organs, including peritoneal dissemination (23 patients, 60.5%), liver metastasis (10 patients, 26.3%), lung metastasis (7 patients, 18.4%), para-aortic lymph node metastasis (6 patients, 15.8%), and multiple organ metastases (15 patients, 39.5%). The remaining patient who underwent R0 resection could undergo resection because there was only one disseminated nodule, and negative cytology was also confirmed.

The median surgical time was 149.5 min (range, 48–365 min), and the median blood loss was 180 ml (range, 5–6100 ml). Postoperative complications occurred in four patients. One patient had bowel obstruction and a urinary tract infection, while the other patients had bowel obstruction, duodenal ulcers, and intra-abdominal abscesses. All complications were grade <IIIa according to the Clavien–Dindo classification.

### Overall outcomes

**Fig. 1:**
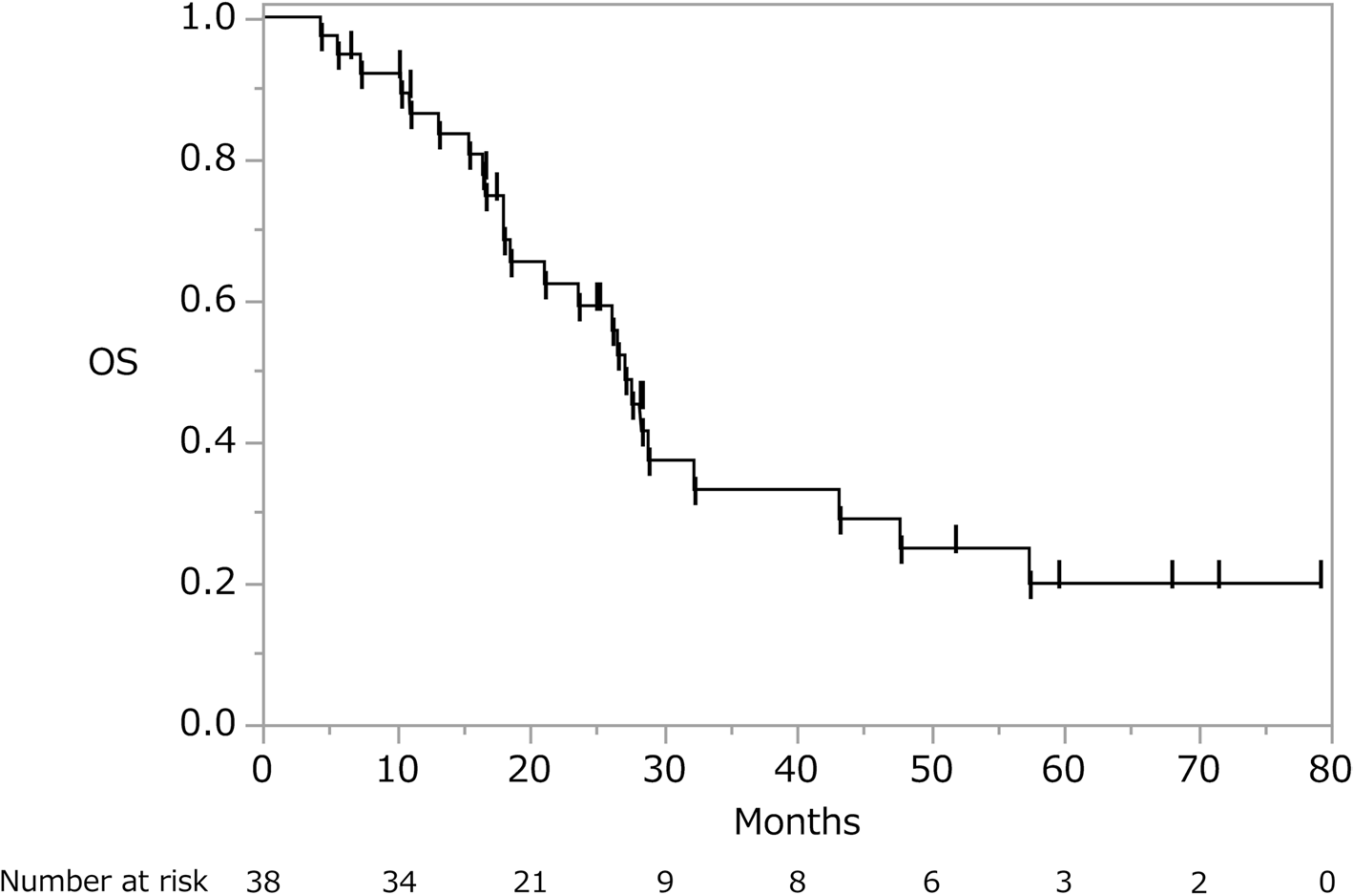
Kaplan–Meier analysis of overall survival in 38 patients undergoing resection of ovarian metastases from colorectal cancer. OS, overall survival

The median follow-up period after the resection of ovarian metastasis was 59.6 months (95% confidence interval [95% CI], 28.2–71.5 months; Kaplan–Meier estimate). The 5-year overall survival rate was 19.9% and the MST was 27.2 months (95% CI, 18.6–43.2 months) (Fig. 1). Five of the eight patients in whom R0 resection was performed experienced recurrence within a median time of 4.6 months (range, 1.4–51.0 months). Eight patients survived > 3 years after ovarian resection, 14 patients were alive, and 3 were disease-free.

**Fig. 2:**
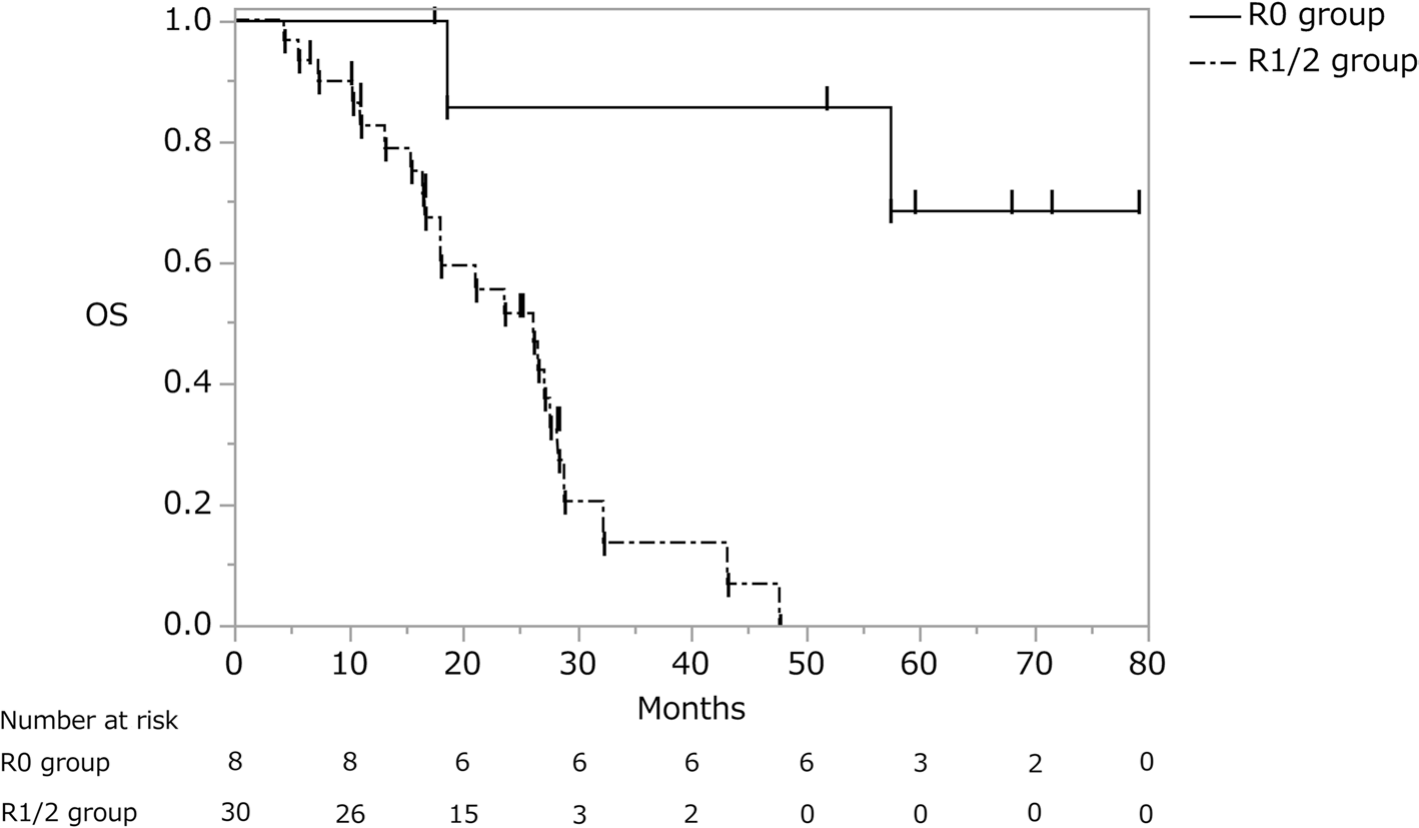
Overall survival curve of the R0 and R1/2 groups. OS, overall survival

The 5-year overall survival rate of patients treated by R0 resection was 68.6%, and the MST was not reached (NR). In the group of patients who underwent R1/2 resection, the 5-year overall survival rate was 0% and the MST was 26.2 months (95% CI, 16.7–28.4 months) (Fig. 2).

The 5-year overall survival rate of patients without extra-ovarian metastases was 68.6%, and the MST was NR. In the group of patients with extra-ovarian metastases, the 5-year overall survival rate was 0% and the MST was 26.2 months (95% CI, 16.2–28.4 months).

The 5-year overall survival rate of patients without perineal dissemination was 41.6%, and the MST was 57.4 months (95% CI, 18.1 months to NR). In the group of patients with perineal dissemination, the 5-year overall survival rate was 0% and the MST was 26.6 months (95% CI, 16.7–28.9 months).

In addition, the operation time of ovarian resection was identified as a prognostic factor. In the group of patients with an operation time of > 170 min, the 5-year survival rate was 11.8% and the MST was 16.5 months (95% CI, 11.1–27.2 months). However, in the group of patients with an operation time of < 169 min, the 5-year survival rate was 22.7% and the MST was 28.9 months (95% CI, 23.7–57.4 months).

The prognosis was not influenced by age, presenting symptoms, menopausal status, chronological status of the metastases, tumor location or histology, depth of invasion, lymph node metastasis, staging of the primary tumor, or the size or bilateral presence of the metastases (Table 1).

**Table 1:** Clinicopathological characteristic and univariate analysis of prognostic factors in the 38 study patients

	No. of patients (%)	MST (months)	*P*-value
Age			0.484
<49 years	16 (42.1)	23.7	
>50 years	22 (57.9)	27.2	
Menstrual status			0.061
Pre-menopausal	10 (26.3)	57.4	
Post-menopausal	28 (73.7)	26.6	
Symptoms			0.845
Yes	19 (50.0)	27.2	
No	19 (50.0)	26.2	
Location			0.247
Proximal colon	12 (31.6)	21.1	
Distal colon	19 (50.0)	28.4	
Rectum	7 (18.4)	27.7	
Differentiation			0.962
Well or moderate	31 (81.6)	27.2	
Poor or mucinous	4 (10.5)	18.1	
Depth of invasion			0.377
T2–3	12 (31.6)	47.8	
T4	24 (63.2)	27.2	
Lymph node metastasis			0.358
Positive	29 (76.3)	26.6	
Negative	9 (23.7)	28.9	
Timing of metastasis			0.775
Synchronous	9 (23.7)	27.8	
Metachronous	29 (76.3)	27.2	
Tumor size			0.663
<12.0 cm	15 (39.5)	28.9	
>12.1 cm	23 (60.5)	26.6	
Side of metastasis			0.125
Unilateral	24 (63.2)	28.4	
Bilateral	14 (36.8)	23.7	
Peritoneal dissemination			0.040
Yes	23 (60.5)	26.6	
No	15 (39.5)	57.4	
Combined metastasis			0.0005
Ovarian metastasis only	7 (18.4)	Not reached	
Other metastases	31 (81.6)	26.2	
Operation time			0.019
<169 min	25 (65.8)	28.9	
>170 min	13 (34.2)	16.5	
Complete resection			0.0004
R0	8 (21.1)	Not reached	
R1/2	30 (78.9)	26.2	

*MST* median survival time

### Preoperative and postoperative chemotherapy

Nine patients with synchronous ovarian metastasis underwent ovarian resection at the time of primary resection. None of the patients received preoperative chemotherapy. Because all patients had unresectable distant metastases such as peritoneal dissemination or that to the liver, lungs, and para-aortic lymph nodes, postoperative chemotherapy was administered to seven patients except one who refused and one who could not receive it because of their age.

**Fig. 3:**
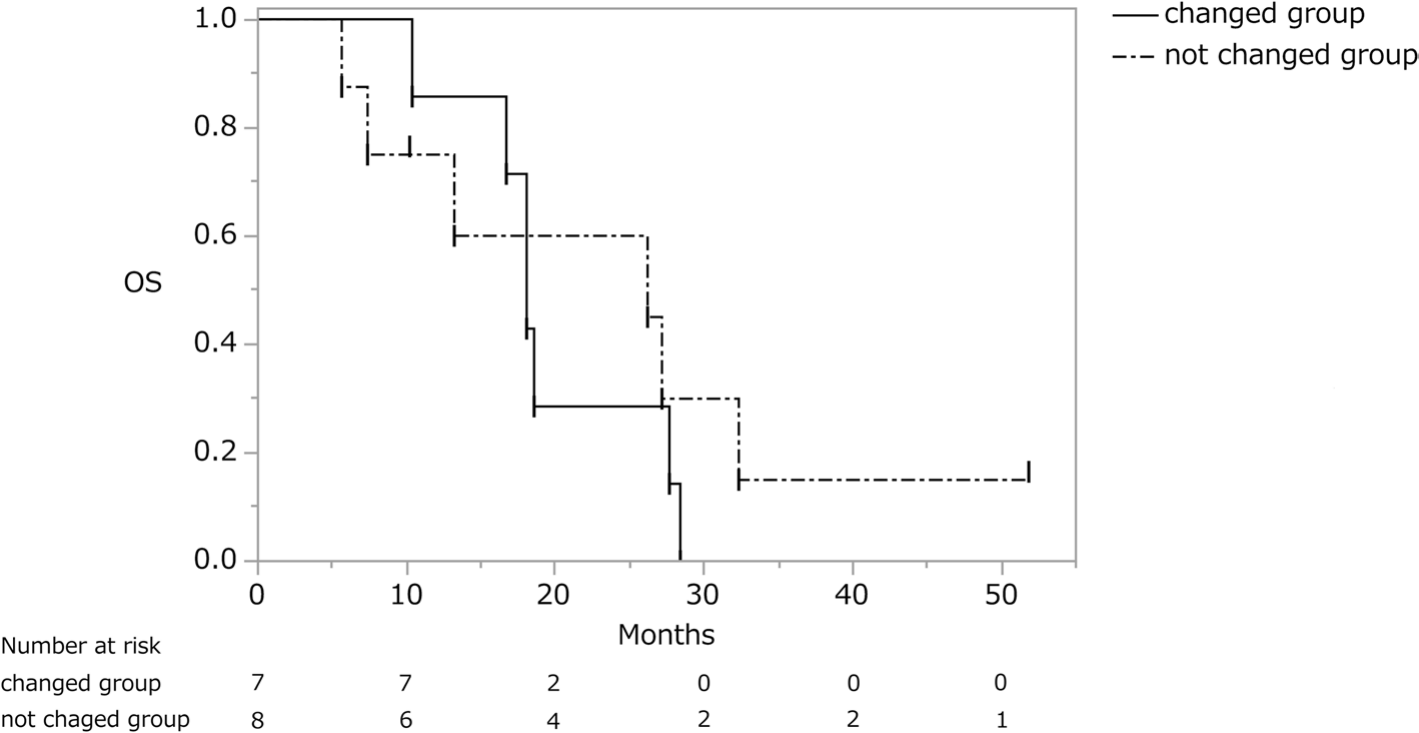
Overall survival curve of patients who resumed chemotherapy with and without a change to their regimen. OS, overall survival

**Fig. 4:**
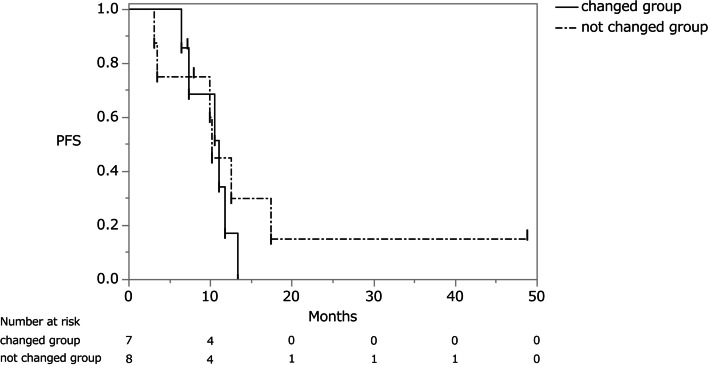
Progression-free survival curve of patients who resumed chemotherapy with and without a change to their regimen. PFS, progression-free survival

**Fig. 5:**
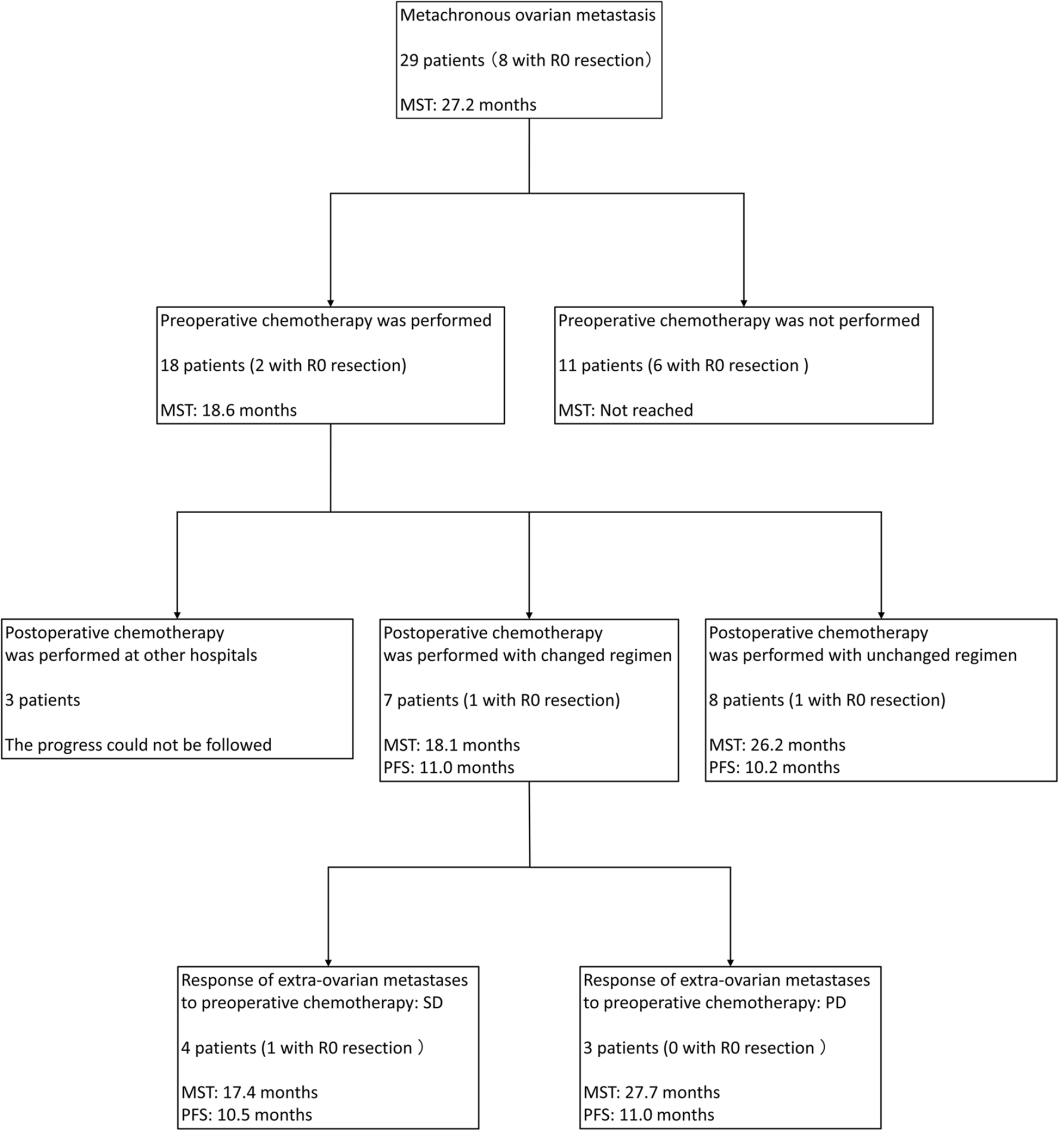
Breakdown and outcome of preoperative and postoperative chemotherapy in patients with metachronous ovarian metastasis. MST, median survival time; PFS, progression-free survival; SD, stable disease; PD, progressive disease

Twenty-eight of 29 patients with metachronous ovarian metastasis received postoperative chemotherapy after ovarian resection (1 patient refused). Of these 28 patients, 18 patients received chemotherapy until immediately before ovarian resection for other metastases. There were eight patients in the first-line, seven patients in the second-line, and three patients in the third-line regimen. Three patients underwent postoperative chemotherapy at other hospitals and their progress could therefore not be followed. The same chemotherapy regimen as that prior to ovarian resection was administered to 8 of the remaining 15 patients (Table 2). The responses to preoperative chemotherapy for extra-ovarian metastasis were partial response in three patients and stable disease (SD) in five patients. Their MST and PFS after ovarian resection were 26.2 months (95% CI, 5.63–32.3 months) and 10.2 months (95% CI, 3.1–17.4 months), respectively (Figs. 3 and 4). Among the eight patients with the same chemotherapy regimen, postoperative chemotherapy was discontinued because of progression of residual metastasis in six patients, whereas in the other two patients it was maintained. In the seven patients with a chemotherapy regimen that was changed after ovarian resection, the responses to preoperative chemotherapy for extra-ovarian metastasis were progressive disease (PD) in three patients and SD in four patients. Of the four patients with SD, however, the postoperative chemotherapy regimen was changed because of an adverse event in one patient and the attending physician’s decision in three patients. Their MST was 18.1 months (95% CI, 10.4–27.7 months) and their PFS was 11.0 months (95% CI, 6.4–13.3 months) (Figs. 3, 4, and 5). In this group, postoperative chemotherapy was discontinued because of progression of the residual metastasis in six patients and an adverse event in one patient. The difference between the two groups was not statistically significant (PFS and MST: *P* = 0.52 and *P* = 0.48, respectively).

**Table 2:** Characteristics of chemotherapy groups

	Changed regimen group	Unchanged regimen group
No. of patients	7	8
Age	58 (range, 25–67)	55 (range, 44–66)
No. of complete resection	1	1
Preoperative chemotherapy period (months)	23 (range, 2–49)	16 (range, 7–52)
No. of preoperative chemotherapy regimens (1^st^/2^nd^/3^rd^)	3/3/1	3/3/2
No. of metastatic organs excluding ovarian metastases (3 organs/2 organs/1 organ/none)	1/2/3/1	0/5/2/1
Responses of extra-ovarian metastases to pre-operative chemotherapy (CR/PR/SD/PD)	0/0/4/3	0/3/5/0
Progression-free survival (months)	11.0	10.2
Median survival time (months)	18.1	26.2

*CR* complete response, *PR* partial response, *SD* stable disease, *PD* progressive disease

## Discussion

Given the poor prognosis of patients with ovarian metastases, some authors of reports published before 2000 proposed only palliative surgical resection, especially when extensive intra-abdominal disease or distant metastasis is present [4–6]. However, a recent study revealed a better prognosis after complete ovarian metastasis resection or cytoreductive surgery [7–17]. In our series, the 5-year overall survival rate of patients treated by R0 resection was 68.6%, versus 0% in those who underwent R1/2 resection. R0 resection of the metastatic site was associated with a significant increase in survival (*P* = 0.0004). These results are similar to the findings of recent studies that revealed a significantly longer 5-year overall survival rate of patients undergoing curative rather than non-curative resection [7–13].

Our study also suggests a better prognosis in patients with isolated ovarian metastasis from CRC resection than in patients with metastases elsewhere. Seven patients who underwent R0 resection were patients with isolated ovarian metastasis, and the other was a patient with localized resectable peritoneal dissemination. Therefore, overall survival of the isolated ovarian metastasis group was almost the same as that of the R0 resection group. The 5-year overall survival rate of the isolated ovarian metastasis group was 68.6%, which is higher than that reported for patients who underwent resection of isolated hepatic metastasis (43.2–59.6%) [18, 19] or isolated pulmonary metastasis (50.9–62.9%) [20–23].

Erroi et al. [7] and Fujiwara et al. [9] reported a 5-year overall survival rate of 77.9 to 80.0% in patients without peritoneal dissemination who underwent ovarian metastasis resection. However, in our series, the 5-year overall survival rate of similar patients was only 41.6%, with none of the patients with peritoneal dissemination surviving for 5 years. Nonetheless, the association between peritoneal dissemination and overall survival was significant (*P* = 0.040). R0 resection is may improve the prognosis; however, it is also important to minimize related complications. McCormick et al. [13] reported that optimal cytoreduction is associated with prolonged PFS and overall survival in patients with metastases, whether localized to the ovaries or widespread. However, high complication rates with a perioperative mortality rate of 5% and significant morbidity of 10% have also been reported [13]. Xu et al. [10] reported a high complication rate and little survival benefit in patients with extended metastases beyond the pelvis who underwent surgical resection and concluded that care must be taken when selecting candidates for surgical resection. In contrast, we encountered no perioperative mortality or significant morbidity in our series. Additionally, the prognosis was worse because of the prolonged operation time (*P* = 0.019). Our results suggest that it is more important to avoid perioperative complications and resume chemotherapy immediately than it is to achieve R0 resection with its associated perioperative complications. For this reason, preoperative patient selection and intraoperative choice of the optimal procedure are very important. Chen et al. [11] have proposed a new scoring system for informing preoperative and intraoperative decision-making by identifying patients who are unlikely to gain survival benefit from surgical resection. Future treatment strategies for patients with ovarian metastases from CRC should include careful selection of patients who are likely to benefit from surgical resection.

In patients being treated for CRC, and especially in those with distant metastasis, an important concern is the optimal combination of chemotherapy and surgical resection. The response to chemotherapy is lower for ovarian metastases than for metastases at other sites [2, 3]. Goere et al. [3] reported the progression of already established metastases or the occurrence of new ovarian metastases during chemotherapy in 87% of patients, whereas a response or the stabilization of extra-ovarian metastases occurred in 65%. Hence, the ovary may provide a “sanctuary” for metastatic cells, although the reasons for its chemoresistance are unknown. However, ovarian metastases are often voluminous, cystic, and mucin-rich, which may reduce their chemosensitivity compared with extra-ovarian metastases. Moreover, because of their chemoresistance, ovarian metastases may rapidly expand, resulting in abdominal distension, pain, or other symptoms. Despite close follow-up of our patients by semi-annual CT, ovarian metastasis was detected in half of the patients only after symptom appearance. The median size of the ovarian metastases was 12.0 cm (range, 2–41 cm). Because expanding ovarian metastases are not likely to be controllable with chemotherapy, their urgent surgical resection should be considered in patients presenting with symptoms.

Chemotherapy can be resumed after the surgical resection of ovarian metastasis, even in patients with PD. In general, the progression of established metastases or the occurrence of new ovarian metastases during chemotherapy are regarded as PD and the chemotherapy regimen is therefore often changed. Yet, this strategy is questionable given that the ovary, as a “sanctuary” site, is likely to be less responsive to chemotherapy [3]. In particular, to our knowledge, no reports have investigated the effect of the choice to resume chemotherapy after resection on the prognosis. In this series, the PFS of patients who resumed the preoperative chemotherapy regimen was 10.2 months, versus 11.0 months in those administered a different regimen. The difference between the two groups was not significant (*P* = 0.52), nor was the difference in their MST (26.2 vs. 18.1 months, respectively; *P* = 0.48). Although there was no significant difference in prognosis between the two groups, it is important to preserve additional chemotherapeutic options because they may contribute to prolonging survival. In this series, one of the patients who resumed the preoperative chemotherapy regimen was able to continue the same chemotherapy regimen for approximately 48 months. However, our conclusions remain to be confirmed in a larger patient population. In this study, there was no significant difference in the comparison of the effects of changes in postoperative chemotherapy on the prognosis, and the treatment strategy could not be determined. However, the data suggested that preserving the next line of chemotherapy may result in a better prognosis.

Finally, the present study was associated with two limitations. First, our study was retrospective in nature and was thus subject to bias. In particular, there may be a selection bias in the attending physician’s decisions regarding chemotherapy for patients whose preoperative chemotherapy response to extra-ovarian metastasis was SD. Second, the study population was relatively small. Thus, these results must be confirmed in a large-scale study.

## Conclusion

The results of this study showed that patients undergoing R0 resection of ovarian metastases from CRC have longer overall survival than patients treated by R1/2 resection, and a poor prognosis was associated with the presence of peritoneal dissemination. Additionally, patients with isolated ovarian metastasis have a better prognosis than those with liver or lung metastases. Our findings also suggest that no change in the chemotherapy regimen following surgery is warranted. This strategy has the additional benefit of preserving the next line of chemotherapy, which may ultimately improve the prognosis. However, the number of patients in this study was small and could not show the statistical differences that underpin this claim. We hope that further evidence will be established by subsequent accumulation of cases and larger investigations. This will further advance the treatment strategy for CRC, which is, the leading cause of death in women.

## Data Availability

The datasets used and/or analyzed during the current study are available from the corresponding author on reasonable request.

## Abbreviations

CRC: Colorectal cancer
MST: Median survival time
PFS: Progression-free survival
CT: Computed tomography
RECIST: Response Evaluation Criteria in Solid Tumors
95% CI: 95% confidence interval
NR: Not reached
SD: Stable disease
PD: Progressive disease
